# Four Types of Hardship and the Moderating Effect of Social Support on Depressive Symptoms in Older Korean Adults

**DOI:** 10.1093/geroni/igaf122.4124

**Published:** 2025-12-31

**Authors:** Meeryoung Kim

**Affiliations:** Daegu University, Daegu, Republic of Korea

## Abstract

Older adults face challenges such as physical frailty, functional impairment, and loneliness. They also face a decline in formal roles after retirement. These challenges can affect their mental health. Pearlin et al.’s (1981) stress theory explains the effects of social support. Long-standing research has argued that social support has both direct and moderating effects depending on the type of challenges older adults face and the availability and use of social support. This study examined the main and moderating effects of social support. This study used panel data from the ninth(2021), ninth additional(2022), and 10th(2023) of the Korea Retirement Income Study(n = 560, 60+). Hierarchical multiple regressions were used for data analysis. In the first step, depressive symptoms (2021), demographic variables, and four hardships (IADL, economic dependence, loneliness, and absence of role) were included. The likelihood of borrowing when financial hardship occurs was used as a moderator variable. The findings showed that gender had different effects on depressive symptoms. Economic dependence had a different effect on depressive symptoms compared to economic independence. Social participation (leisure activities, class reunions, and community activities) and exercise directly reduced depressive symptoms. Instrumental social support had a moderating effect when experiencing economic hardship. These results suggest that not all difficulties experienced by older adults are stressful. Structural social support had a main effect on reducing depressive symptoms in older adults, while functional social support had a moderating effect. Regardless of the difficulties faced by older adults, social support proved to be an important factor in reducing depressive symptoms.

